# Ultrasound-guided pulsed radiofrequency versus perineural platelet rich plasma injection for the treatment of idiopathic carpal tunnel syndrome: a prospective randomized controlled study

**DOI:** 10.1186/s12871-025-03257-x

**Published:** 2025-08-13

**Authors:** Engi Yousry Hashem, Faten Saeed Shamandy, Ahmed Fawzy Elmulla, Magdy AbdelAziz Mansour

**Affiliations:** Department of Anesthesia and Pain Management, Medical Research Institute, University of Alexandria, 165 El-Horeya Rd, Al Ibrahimeyah Qebli WA Al Hadrah Bahri, Qesm Bab Sharqi, Alexandria, Egypt

**Keywords:** Carpal tunnel syndrome, Platelet-rich plasma, Pulsed radiofrequency, Steroids, Boston carpal tunnel questionnaire, Nerve conduction velocity, Median nerve cross-sectional area, Visual analog scale

## Abstract

**Background:**

Carpal tunnel syndrome (CTS) is the most common focal mononeuropathy caused by the compression of the median nerve within the carpal tunnel. Ultrasound-guided hydrodissection with corticosteroids, platelet-rich plasma injection, and median nerve pulsed radiofrequency are all potential treatments for relieving symptoms of CTS in mild and moderate cases, comparison between their clinical outcomes is yet to be studied.The primary outcome was pain measured by Visual Analog Scale (VAS). The secondary outcomes included functional outcome evaluated by the Boston Carpal Tunnel Questionnaire (BCTQ), Nerve Conduction Velocity (NCV) and median nerve cross-sectional area (CSA).

**Methods:**

This prospective, double-blinded, randomized controlled study was conducted on Seventy-five patients diagnosed with mild to moderate CTS, they were randomly allocated into three equal groups. The control group received a median nerve perineural injection of bupivacaine with methylprednisolone. The PRF group received median nerve pulsed radiofrequency in addition to bupivacaine. Finally, the PRP group received a perineural injection of platelet-rich plasma.

**Measurements:**

Were conducted at specific time intervals: before the intervention, at one week, two months, and four months post-intervention for VAS, BCTQ, and CSA. NCV was only evaluated before the intervention and after four months.

**Results:**

All patients experienced a statistically significant improvement in pain, symptoms, function, CSA of the median nerve and NCV following intervention. The PRF and steroid groups exhibited greater improvements than the PRP group. The PRP group showed the least improvement compared to the other two groups.

**Limitations:**

Short study period, single centred study.

**Conclusions:**

The current study suggests that ultrasound-guided hydrodissection of the median nerve, with local anesthetic/steroids or PRP and PRF are effective in alleviating pain and improving the functional outcome. Nevertheless, it was revealed that PRF and steroid injection were more efficacious in enhancing short term functional outcomes compared to PRP injection.

**Trial registration:**

The study was retrospectively registered in the “Clinical Trials Library for Protocol Registration and Results System” under NCT05053477 on September 11, 2021.

**Supplementary Information:**

The online version contains supplementary material available at 10.1186/s12871-025-03257-x.

## Introduction

Carpal tunnel syndrome (CTS) is a compression neuropathy affecting the median nerve at the wrist. The pathophysiology of CTS involves increased endoneurial pressure, reduced neuronal microcirculation, neural ischemia, edema, and fibrosis [1]. Nonsurgical treatments such as splints and physical therapy are recommended for mild-to-moderate CTS but have limited therapeutic effects [2].

Ultrasound-guided hydrodissection, corticosteroids, platelet-rich plasma (PRP) injections, and pulsed radiofrequency (PRF) treatment are all potential therapies. The precise mechanisms underlying the therapeutic benefits of such treatments remain incompletely understood. Hydrodissection can reduce gliding resistance in patients with CTS, as it creates a fluid plane around the nerve and dislodges perineural adhesions [3]. Corticosteroids alleviate inflammation by modifying vascular wall permeability [4]; nonetheless, the mechanism through which they confer analgesic benefits is likely attributable to their antifibrotic properties [5].

Platelet-rich plasma (PRP) therapies aim to increase self-healing by using autologous growth factors to stimulate wound healing, angiogenesis, and axonal regeneration [4].PRP products exhibit significant therapeutic potential as neuroprotective, neurogenic, and neuroinflammatory modulators. They are employed in the management of peripheral nerve injuries and neuropathies. Plasma rich in growth factors encompasses a diverse array of growth factors, microparticles, and bioactive mediators. Upon the infiltration of PRP, cell signaling molecules, such as neurotrophic factors—including nerve growth factor (NGF), brain-derived neurotrophic factor (BDNF), platelet-derived growth factor (PDGF), and insulin-like growth factor 1 (IGF-1)—as well as neurotropic factors (fibrin, fibronectin, and vitronectin) are released. These elements facilitate the modulation of early inflammation, the activation of stem cells, macrophage polarization, the resolution of inflammation, angiogenesis, and fibrogenesis. Consequently, they are indispensable for the functional recovery of nerve tissue(6).

Pulsed radiofrequency (PRF) treatment alleviates pain by delivering electric impulses and heat bursts at a temperature of less than 42 °C [5]. PRF selectively regulates small-diameter Aδ and C-type fibers through modifications to mitochondria, microfilaments, and microtubules within these fibers [7].PRF techniques are routinely employed for their neuromodulatory effects on resting baseline pain perception, while concurrently mitigating the risks associated with thermodestructive lesioning. Despite a limited understanding of the underlying mechanisms, the application of electrical fields has demonstrated the capacity to exert reversible effects on impulse transmission along small unmyelinated fibers. This phenomenon can induce alterations in the excitatory C and Aδ fibers, which are pivotal for the transmission of nociceptive and neuropathic pain. Several mechanisms have been postulated, including modifications at nerve synapses and alterations in nerve permeability induced by the electrical field. Furthermore, changes in c-Fos signaling pathways may augment descending noradrenergic and serotonergic inhibitory pathways [8].

The aim of the study is to evaluate the role of pulsed radiofrequency versus platelet rich plasma injection in treatment of idiopathic mild to moderate carpal tunnel syndrome.

### Study registration

The study was retrospectively registered in the “Clinical Trials Library for Protocol Registration and Results System” under NCT05053477 0n September 11, 2021. This investigation strictly followed CONSORT requirements as outlined in their statement published in 2010 [9].

## Methods

This double-blind, randomized controlled trial was conducted on 75 patients recruited from the pain clinic at the Medical Research Institute, Alexandria University, who were diagnosed with mild to moderate idiopathic carpal tunnel syndrome (CTS) for more than 3 months and who had not responded positively to conservative treatment methods.

After declaring the research benefits and potential side effects, a comprehensive Informed written consent form was obtained from all participants for their involvement in the study. Additionally, written informed consent was acquired from the patients for the publication of the ultrasound images from the study.

### Assessment and preparation

**Fig. 1:**
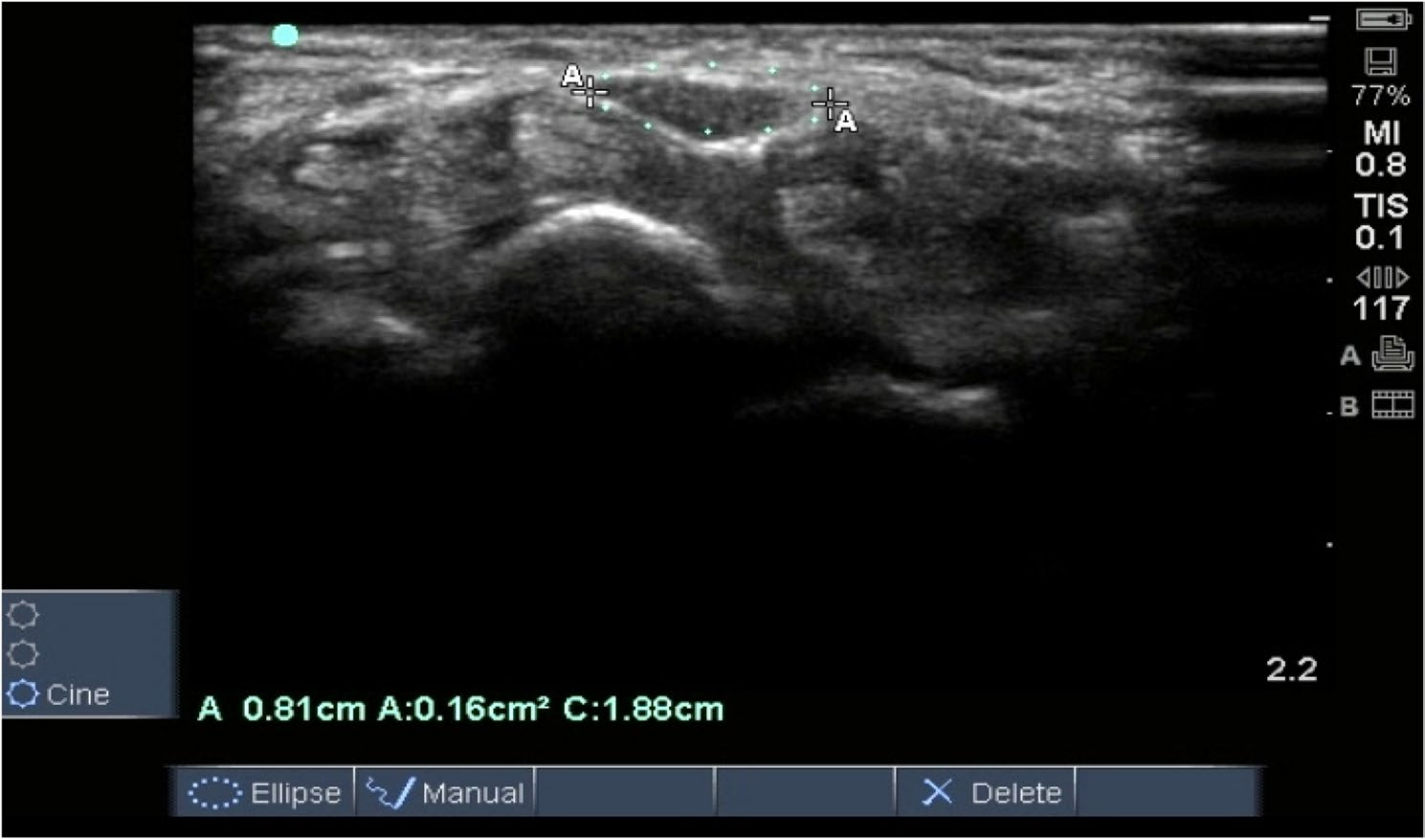
CSA of median nerve measurement at level of pisiform bone
  - Any secondary causes for carpal tunnel syndrome were identified including and not restricted to (tenosynovitis. Ganglion cyst, tumor or space occupying lesions, bifid nerve median or a patent median artery, ect).
- Nerve conduction velocity study (SNCV) were conducted by the same physiotherapist within the same laboratory for all the patients pre and post procedural.
- Laboratory investigations via venous blood sample: 
  - Complete blood picture.
  - Platelet count.
  - Random blood sugar.
  - Prothrombin activity (PT, INR).

Patients were thoroughly assessed by:

- Detailed medical and surgical history taking.
- Clinical examination for median nerve by Reverse Phalen’s test, Tinel’s tests and Boston Carpal Tunnel Syndrome Questionnaire (BCTQ).
- Ultrasound examination:
  - Cross-sectional area (CSA): The median nerve was identified using a 38 mm, L25x,13 − 6 MHz, linear array transducer with a portable, bedside Ultrasound unit (Sonosite S-Nerve, Sonosite Inc.,USA). The participants’ wrists were maintained in neutral positions, scanning was done in short axis, as the probe was parallel to proximal crease of hand where the CSA was measured. The greatest lateral measurements in anteroposterior and lateral dimensions were recorded (mm^2^) and then calculated into an elliptical shape for subsequent calculation of its surface area (mm^2^)and the cross sectional area of the nerve was measured in mm2. (Fig. 1).

### Inclusion criteria

- Patients aged (20–60) years of both gender.
- Patients with mild to moderate idiopathic CTS The grading of CTS was based on Padua’s study as (1) Mild: abnormal, sensory nerve conduction velocity (SNCV) with a normal distal motor latency (DML), (2) Moderate: abnormal SNCV and DML, and (3) Severe: absence of SNCV and abnormal DML [10, 11].
- Patient who are failed to respond to conservative treatment (such as splint, medications, physical therapy) for at least three months.

### Exclusion criteria

- Severe CTS severe CTS (distal latency to abductor pollicis brevis muscle > 6.5 ms or with absent sensory potentials of the median nerve by electrophysiological study [7], cross-sectional area [CSA] of median nerve > 15.0 mm2 by ultrasonography [12].
- Secondary CTS due to systemic disease including thyroid disease, diabetes mellitus, or acromegaly or due to local causes as identified by the pre-procedural Ultrasound including tenosynovitis. Ganglion cyst, tumor or space occupying lesions, bifid nerve median or a patent median artery.
- Other neuropathies overlapping with CTS.
- Previous release surgery for the median nerve or wrist surgery.
- Pregnancy.
- Known allergies to any of the medications or who were on concurrent steroids.
- Previous injections to CTS upto 6 months.

Patients were randomly assigned to one of the three studied groups. The allocation was performed via a computer-generated randomization program (www.randomizer.org) in which patients were equally assigned to the three studied groups.Each patient’s randomization number was concealed until the end of the study from both the patients and the outcome assessor who was blinded to the procedure used.The procedure used for treatment was not revealed to the patients until study completion.

### Intervention

All the procedures were conducted under ultrasound guidance (Sonosite^®^ S-Nerve™ ultrasound machine, FUJIFILM SonoSite, USA) with a 38 mm, L25x, 13–6 MHz, linear array probe. Patients were in a supine position while their forearm was extended with the palm facing up and slightly extended with a small pillow. This position was used during the ultrasound assessment and injection.

**Fig. 2:**
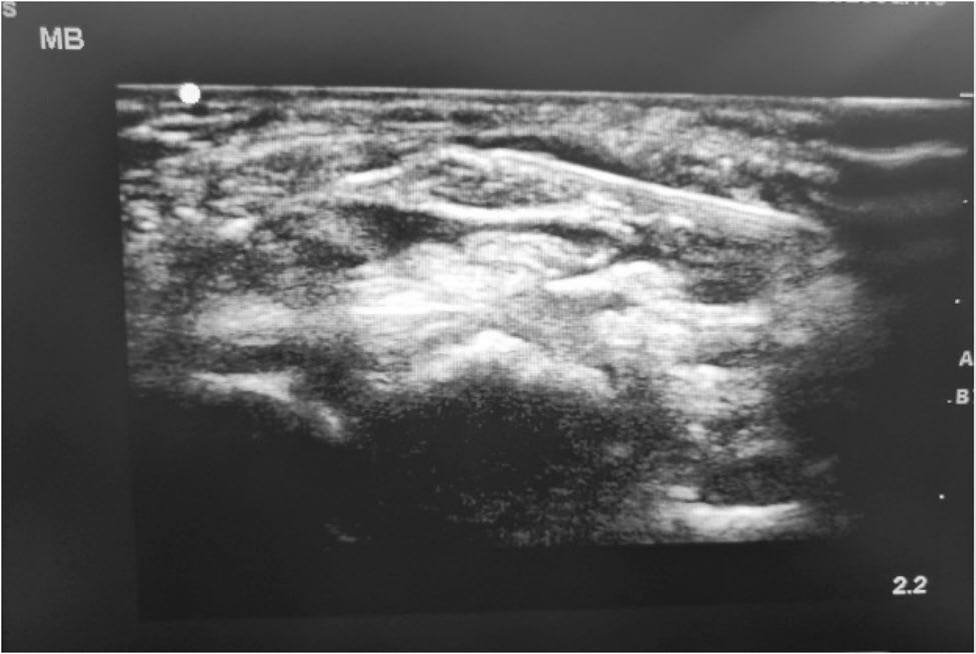
Hydodisection of the median nerve using a 22-gauge needle

- The control group (*n* = 25) received a perineural injection of bupivacaine (0.25%) with methylprednisolone (40 mg) in a total volume of 2 ml. Injection was done to circumstantially Hydro dissecting the nerve from the underlying structures and the retinaculum above. (Fig. 2).

**Fig. 3:**
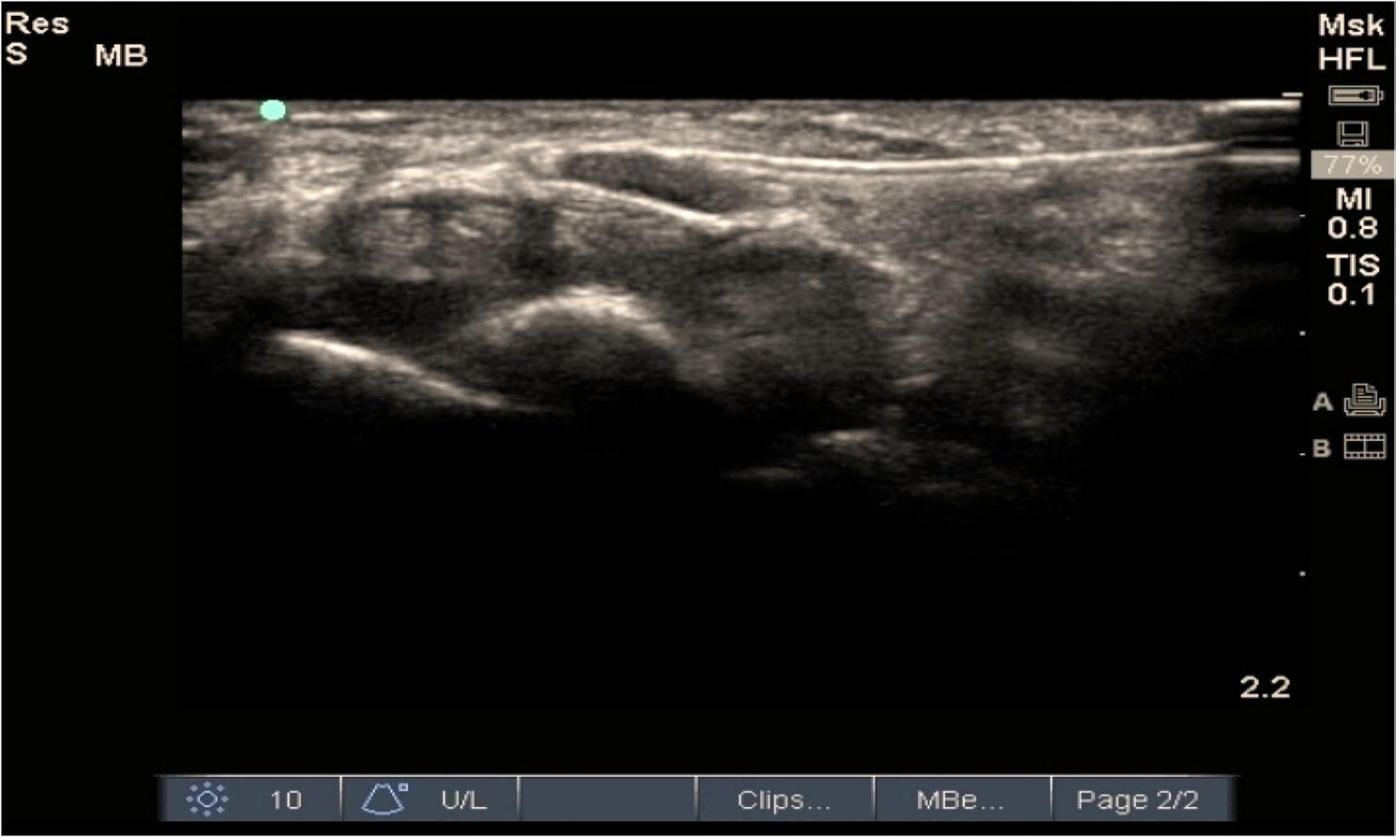
22-gauge, 50-mm radiofrequency cannula inserted above median nerve

The PRF group (n = 25): After the identification of the median nerve using ultrasound, a 5-cm radiofrequency curved cannula with a 10-mm active tip was meticulously advanced towards the median nerve employing the in-plane technique. Utilizing the Neurotherm (NT1000 (NeuroTherm® NT2000Ix, Abbott Medical, USA),), both sensory and motor stimulations were assessed. Sensory responses were elicited at 50 Hz and upto 0.5 V, while motor responses were evaluated at 2 Hz and up to 1 V, respectively. Pulsed radiofrequency (PRF) lesions were applied for a duration of 120 s at a frequency of 2 Hz and a pulse width of 20 ms, voltage 45 v, maintained at a temperature of 42 °C. Subsequently, 2 ml of 0.25% bupivacaine was administered perineurally. (Fig. 3).

Finally, the PRP group (*n* = 25) received a perineural injection of 2 ml of PRP. PRP was prepared by withdrawing 15 ml of fresh whole blood from each patient in citrate tubes. The tubes were initially centrifuged at 3000 rpm for 10 min. The resulting supernatant plasma and buffy coat were then transferred to another sterile plain tube and subjected to hard spin at 4000 rpm for 15 min. The sediment comprising 2 ml of PRP was suspended by gently shaking it, then it was injected perineurally under real-time ultrasound guidance using the inplane technique.

### Post procedure care for three groups

- Patients were advised to apply ice on the injection site at day of intervention and modify activity as tolerated to alleviate any post injection discomfort or pain.
- Pain medication in the form of paracetamol (500 mg) only was allowed for the next 4 months if needed. The patients were instructed to stop analgesics 48 h before visit of follow up to allow proper symptoms assessment.
- Physical therapy, splinting, or exercise were not allowed.

### Study endpoints

The primary outcome measure among the groups was the visual analog scale (VAS). The secondary outcomes included cross-sectional area (CSA), functional outcome as evaluated by the Boston Carpal Tunnel Questionnaire (BCTQ), and an electrophysiological study via nerve conduction velocity (NCV).

### Measurements

Baseline measurements assessing demographic data, CSA (mm^2^), baseline pain and disability through the VAS and BCTQ [13] were obtained before the intervention, followed by subsequent assessments at one week, two months, and four months after the procedure. An Arabic version of the BCTQ was handed to the patients at each visit. NCV testing was carried out by the same physiotherapist in the same laboratory and machine configurations to validate the standardization both initially and at four months. Any complications that occurred during or after any interventions were reported and managed accordingly.

### Statistical analysis

The required sample size of the study was calculated via the PASS Version 20 program. To ascertain the proportional difference in pain relief assessed by the visual analog scale (VAS) compared with the control group, a minimum hypothesized total sample size of 75 adults (25 per group) is necessary. This computation incorporates a 95% confidence interval and 80% statistical power, utilizing the chi-square test [9]. The data were analyzed via the IBM SPSS software package version 20.0 (Armonk, NY: IBM Corp). Qualitative data are expressed in terms of numbers and percentages. The normality of the distribution was confirmed by the Shapiro‒Wilk test. Quantitative data are presented as the means, standard deviations, medians, and interquartile ranges (IQRs). Statistical significance was considered at the 5% level. For categorical variables, a chi-square or Monte Carlo test was conducted to compare various groups. To compare normally distributed quantitative variables among more than two groups, an F test (ANOVA) was performed, while pairwise comparisons were performed through post hoc testing. The Kruskal‒Wallis test was used for non normally distributed quantitative variables with multiple studied groups, where pairwise comparisons took place via Dunn’s multiple comparisons test under post hoc analysis.

## Results

**Fig. 4:**
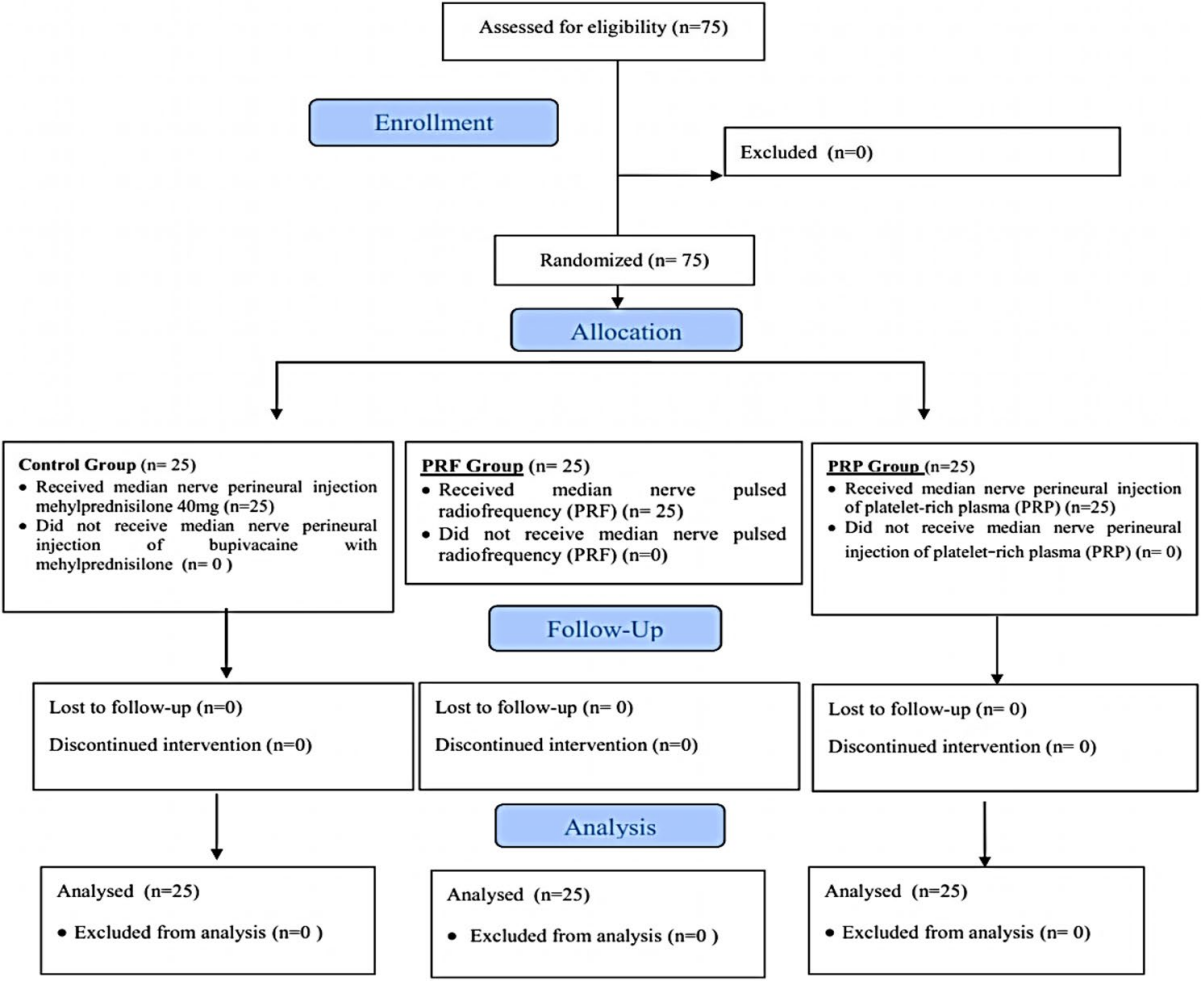
CONSORT flow diagram of the studied participants

Seventy-five patients were recruited, and none of them were excluded. The 75 patients who fulfilled the inclusion criteria were randomized into 3 equal groups of 25 patients each. The attrition rate was 0% (Fig. 4).

Demographic data were comparable across the three study groups, with no statistically significant differences among the three groups (Table 1).

**Table 1:** Comparison between the three studied groups regarding the demographic data

	PRF (n = 25)	PRP (n = 25)	Control (n = 25)	p
Sex (females)	25 (100%)	25 (100%)	25 (100%)	–
Age (years)	33.36 ± 7.78	34.08 ± 7.5	35.36 ± 8.78	0.674

Data were expressed using Mean ± SD

*PRF* pulsed radiofrequency, *PRP* platelet rich plasma

Standard deviation. *P* < 0.05 is considered significant

There was no statistically significant difference among the three groups prior to the intervention concerning the various clinical criteria, including VAS, CSA, both components of the BCTQ and the different parameters of the nerve conduction tests (Table 2).

**Table 2:** Comparison between the three studied groups regarding clinical criteria before the intervention

Preintervention		RF		PRP	Control	(*p*)
		(*n* = 25)		(*n* = 25)	(*n* = 25)	
VAS						0.762
Min. – Max.		6–10		7–10	5–10	
Median (IQR)		8 (7–9)		8 (8–9)	8 (8–9)	
CSA (mm2)						0.577
Min. – Max.		10.20–14.60		9.60–15.0	9.80–15.0	
Median		12.4		12	12	
BCTQ (SSS)						0.072
Min. – Max.		3.60–5.0		3.50–5.0	3.20–5.0	
Median (IQR)		4.7(4.10–5.0)		4.7(4.20–5.0)	4.1(3.70–4.60)	
BCTQ (FSS)						
Min. – Max.		3.20–5.0		3.70–4.90	3.10–5.0	0.569
Median(IQR)		4.3(4.0–4.50)		4.1(4.0–4.70)	4.1(4.0–4.50)	
Distal motor latency (msec)						
Min. – Max.		4.30–5.20		4.30–5.20	4.30–5.20	(0.956)
Median		4.70		4.70	4.70	
Sensory conduction velocity (m/s)						
Min. – Max.		35.10–37.80		35.10–37.30	35.10–38.40	(0.195)
Median		36.40		35.80	36.30	
Distal CMAP amplitude (mV)						
Min. – Max.		6.80–10.10		7.10–10.40	6.80–10.0	(0.491)
Median		8.80		8.70	8.50	

Data were expressed using Mean ± SD

*PRF* pulsed radiofrequency, *PRP* platelet rich plasma

Standard deviation. *P* < 0.05 is considered significant

The group analysis unveiled a significant reduction in all groups in the Visual Analog Scale (VAS) score pre and post-intervention at one week, two months, and four months.

**Fig. 5:**
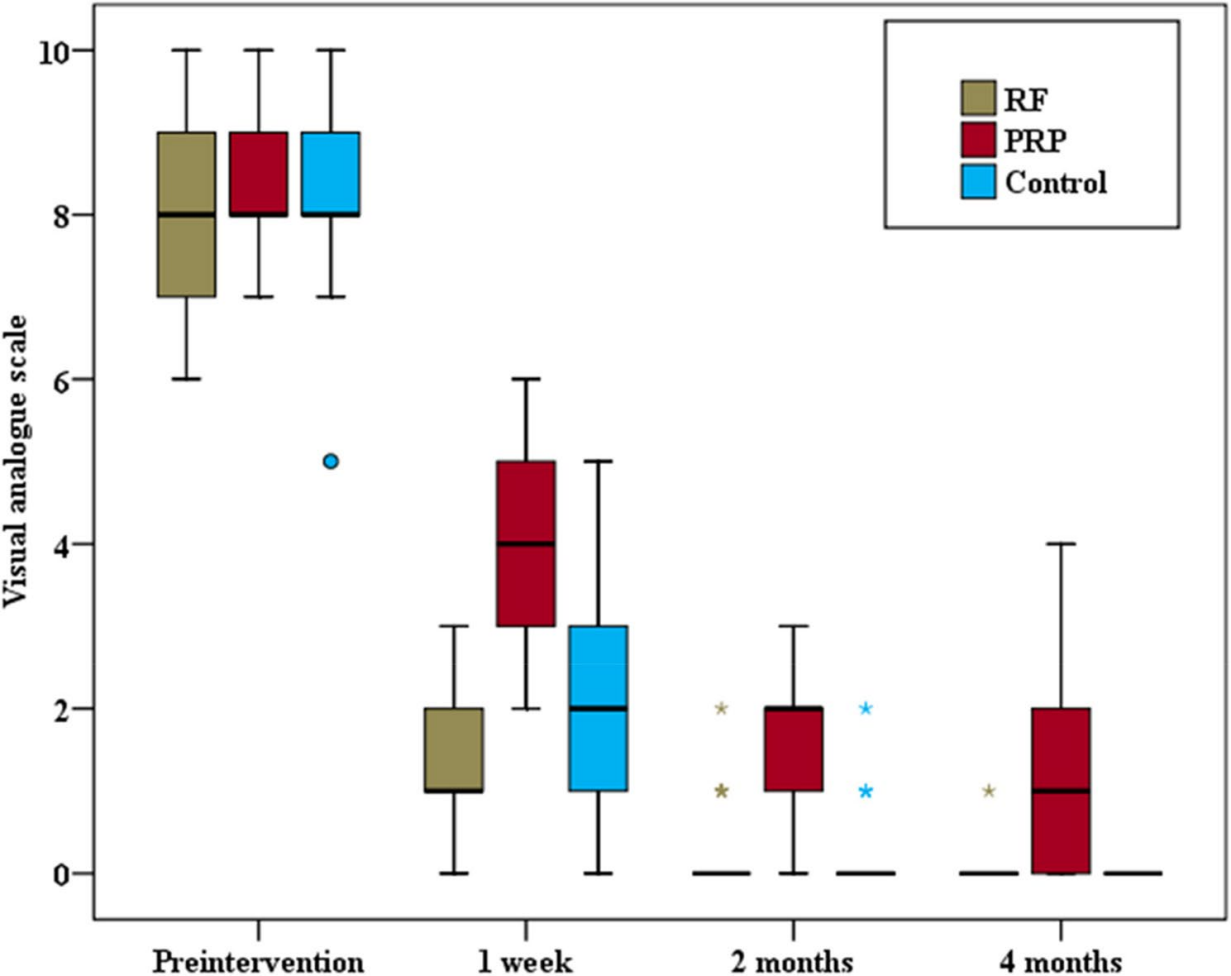
Comparison between the three studied groups as regards Visual Analogue scale

The VAS score was significantly different among the three groups at different follow-up times: one week (*p* < 0.001), two months (*p* < 0.001), and four months (*p* < 0.001). Pairwise comparisons between the two groups in terms of the VAS score at different study times revealed statistically significant differences between the PRF group and the PRP group after one week (*p* < 0.001), after two months (*p* < 0.001), and after four months of intervention (*p* < 0.001), where lower scores were recorded in the PRF group. Additionally, there was a statistically significant difference in the VAS score at different study times between the steroid group and the PRP group after one week (*p* < 0.001), after two months (*p* < 0.001), and after four months of intervention (*p* < 0.001), where lower VAS scores were recorded in the steroid groups. However, there was no statistically significant difference in the VAS score at different study times between the PRF group and the control group after one week (*p* = 0.079), after two months, (*p* = 0.838), and after four months of intervention (*p* = 0.768) (Fig. 5).

**Fig. 6:**
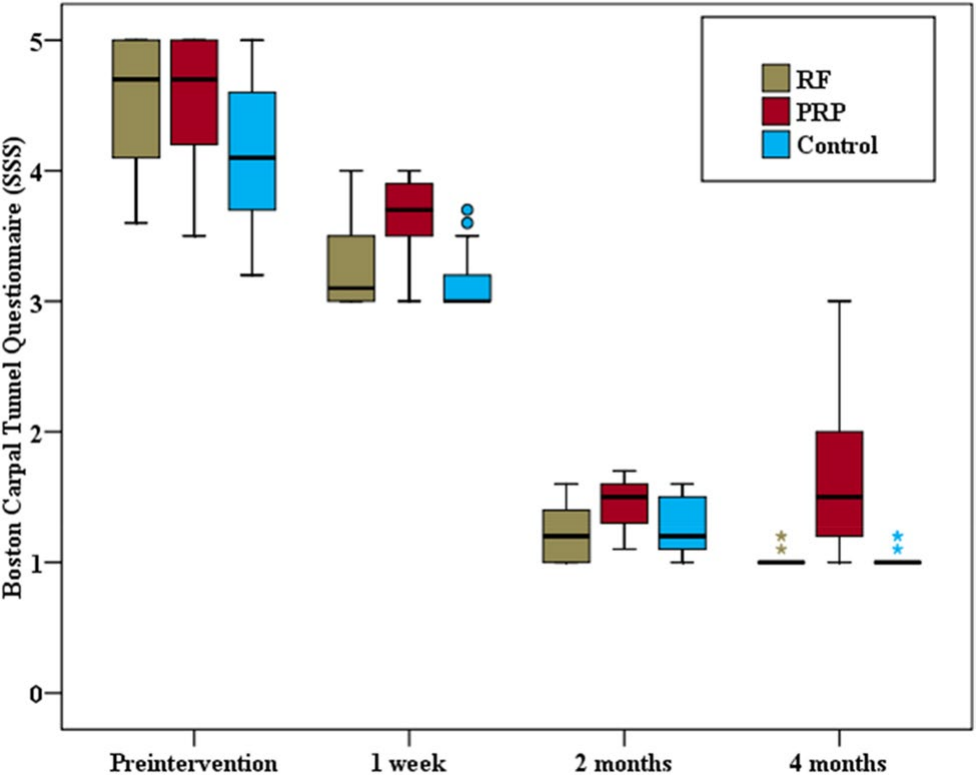
Comparison between the three study groups as regard Boston Carpal Tunnel Questionnaire (SSS)

**Fig. 7:**
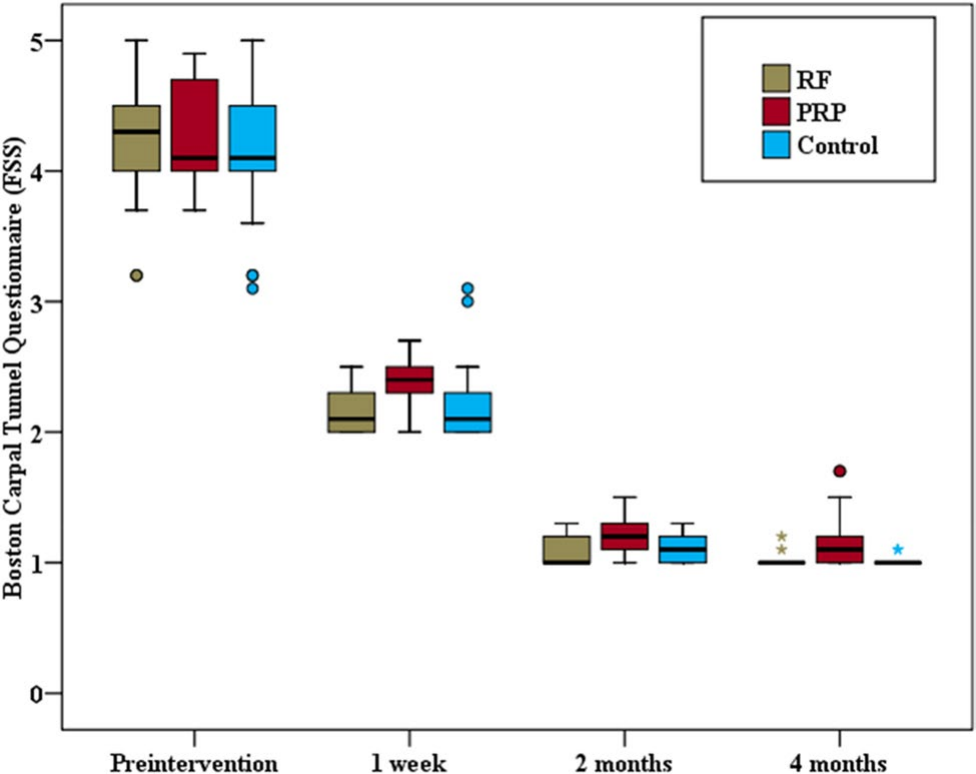
Comparison between the three study groups as regard Boston Carpal Tunnel Questionnaire (FSS)

The same group comparison revealed statistically significant improvements in both components of the BCTQ at all follow-up time points compared with the preintervention readings. A statistically significant difference in BCTQ (SSS) and BCTQ (FSS) scores was detected among the three groups at various follow-up times (*p* < 0.001). Pairwise comparisons between each group revealed significant differences in BCTQ (SSS) and BCTQ (FSS) scores between the PRF and PRP groups at all studied time points (*p* = 0.001). Similarly, significant differences in BCTQ (SSS) and BCTQ (FSS) scores were found between the PRP group and the steroid group at all the studied follow-up times (*p* < 0.001, *p* = 0.002, *p* < 0.001 for the BCTQ (SSS) and *p* < 0.001, p_3_ = 0.001, *p* < 0.001 for the BCTQ (FSS) respectively). Conversely, no statistically significant difference in BCTQ was detected between the PRF group and the control group at any of the studied times. (Figs. 6 and 7).

**Fig. 8:**
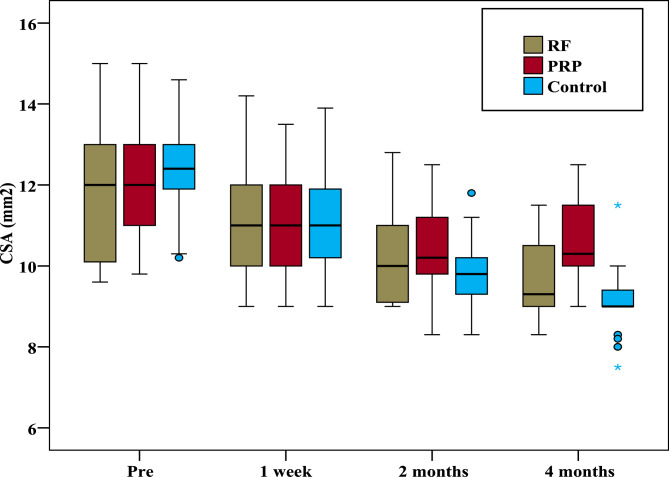
Comparison between the three studied groups regarding median nerve cross sectional area (mm2)

With respect to the CSA, the same group comparison revealed a statistically significant decrease in the median nerve CSA in all the groups at all the studied times compared with the preintervention values. A significant difference among all the groups was recorded only after four months (*p* < 0.001). Pairwise comparisons revealed no significant difference in the CSA between the PRF and steroid groups after four months (*p* = 0.147), but a significant difference was recorded between the steroid and PRP groups (*p* = 0.002) and between the PRF and PRP groups (*p* < 0.001) (Fig. 8).

After four months of intervention, all the groups showed significant improvements in different NCS parameters, including distal motor latency (msec), CMAP amplitude (mV), and sensory conduction velocity (SCV) (m/s). Compared with the PRP group, the steroid group and PRF group demonstrated a significantly greater improvement in all NCSs. The data regarding all the parameters and the degree of significance are presented in (Table 3)

**Table 3:** Comparison between the three study groups regarding NCV (distal motor latency (msec), distal CMAP amplitude (mV) and sensory conduction velocity (m/s)

		RF (*n* = 25)	PRP (*n* = 25)	Control (*n* = 25)	(*p*)	Sig. bet. grps.
Distal motor latency (msec)	Before				(0.956)	–
	Min. – Max.	4.30–5.20	4.30–5.20	4.30–5.20		
	Median	4.70	4.70	4.70		
	4 months					
	Min. – Max.	3.40–4.50	3.40–4.80	3.60–4.50	(< 0.001^*^)	p_1_ < 0.001^*^, p_2_ = 0.284, p_3_ = 0.014^*^
	Median	3.70	4.20	3.90		
	(p_0_)	(< 0.001^*^)	(< 0.001^*^)	(< 0.001^*^)		
Distal CMAP amplitude (mV)	Before					
	Min. – Max.	6.80–10.10	7.10–10.40	6.80–10.0	(0.491)	–
	Median	8.80	8.70	8.50		
	4 months					
	Min. – Max.	9.20–15.50	8.30–14.0	9.10–15.20	(< 0.001^*^)	p_1_ < 0.001^*^, p_2_ = 0.624, p_3_ < 0.001^*^
	Median	13.90	9.80	13.60		
	(p_0_)	(< 0.001^*^)	(< 0.001^*^)	(< 0.001^*^)		
Sensory conduction velocity (m/s)	Before					
	Min. – Max.	35.10–37.80	35.10–37.30	35.10–38.40	(0.195)	–
	Median	36.40	35.80	36.30		
	4 months					
	Min. – Max.	42.30–55.30	36.80–54.20	41.20–53.20	(< 0.001^*^)	p_1_ < 0.001^*^, p_2_ = 0.444, p_3_ = 0.001^*^
	Median	50.90	43.20	49.50		
	t(p_0_)	(< 0.001^*^)	(< 0.001^*^)	(< 0.001^*^)		

Pairwise comparison bet. each 2 groups was done using Post Hoc Test (Tukey)

*PRF* pulsed radiofrequency, *PRP* platelet rich plasma, *Min* minimum, *Max* maximum, *n* numbers

Statistically significant at p ≤ 0.05

p: *p* value for comparing between the three studied groups

p_0_: *p* value for comparing between before and 4 months in each group

p_1_: *p* value for comparing between RF and PRP

p_2_: *p* value for comparing between RF and Control

p_3_: *p* value for comparing between PRP and Control

**Fig. 9:**
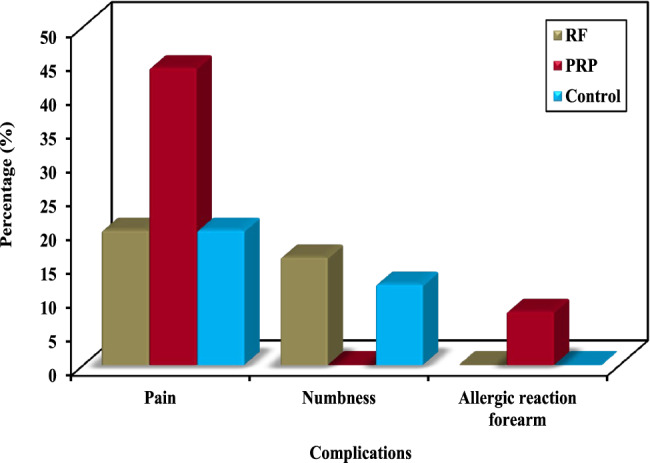
Comparison between the three studied groups according to complications

Notably, PRP was associated with a greater incidence of complications (48%) than RF (24%) and steroids (28%) in terms of procedural pain, numbness, and allergic reactions in the forearm. However, these differences did not reach statistical significance, as indicated by p values of 0.092, 0.151, and 0.331, respectively (Fig. 9).

The percentage of reduction in different parameters among the three groups is illustared in (Table 4).

**Table 4:** Descriptive analysis of the percent of reduction in different parameters in each group

Percent of reduction (%)	PRF (*n* = 25)	PRP (*n* = 25)	Control (*n* = 25)
VAS			
Min. – Max.	85.71–100.0	42.86–100.0	100.0–100.0
Median (IQR)	100.0 (100–100)	88.89 (75–100)	100.0 (100–100)
CSA			
Min. – Max.	6.25–33.33	0.0–25.0	11.76–37.50
Median (IQR)	17.0 (14.0–22.50)	9.73 (5.45–14.17)	25.45 (22.33–29.23)
BCTQ (SSS)			
Min. – Max.	70.73–80.0	28.57–80.0	65.63–80.0
Median (IQR)	76.60 (74.36–80.0)	67.39 (48.72–76.0)	75.61 (72.97–78.26)
BCTQ (FSS)			
Min. – Max.	68.75–80.0	63.83–79.59	67.74–80.0
Median (IQR)	75.61 (73.33–77.78)	72.50 (70.0–75.61)	75.0 (72.97–77.78)

*PRF* pulsed radiofrequency, *PRP *platelet rich plasma

## Discussion

In the current study, US-guided median nerve PRP injection, steroid injection and PRF yielded significant improvements in the VAS score, BCTQ score, and NCV at all follow-ups compared with those at baseline, with comparable improvements between the PRF group and the steroid group after four months and significantly less improvement in the PRP group.

Consistent findings underscore the short-term efficacy of perineural steroid injection treatment [14],it is postulated to attenuate inflammation via the reducion of the expression of acute inflammatory factors such as CRP and IL-6 and edema affecting the nerve and surrounding tissues within the carpal tunnel, thereby diminishing the pressure exerted on the median nerve [15] though recent molecular genetic analyses have challenged this traditional view and have focused on the anti-fibrinolytic properties as reported on the downregulation of fibrosis related genes *Col1A1* (collagen type 1 alpha 1 chin), *Col1A2*, and *Col3A1* in ten patients who underwent ultrasound-guided injection with triamcinolone acetonide [16].

However, divergent reports persist regarding long-term outcomes. Enhanced results are anticipated in cases of milder symptoms, those persisting for less than one year, and less severe electrophysiological findings. A dosage of 40 mg of triamcinolone acetonide is frequently utilized, or its equivalent steroid dosages, with an average injection volume of 3.54 mL recommended for median nerve mobilization, ensuring adequate hydrodisection and superior distribution of the steroid dosage around the nerve. A dosage of 10 mg of triamcinolone acetonide may prove more advantageous in mitigating adverse effects. Ultrasound-guided injection for carpal tunnel syndrome enhances precision, minimizes the risk of intraneural injection, alleviates pain, and augments both efficacy and outcomes in comparison to the traditional landmark technique [17, 18].

The current study posits enhanced short-term to mid term clinical outcomes following PRP injections upto 4 months. There exists a compelling body of evidence supporting the potential of PRP in fostering nerve regeneration, including: neuroprotection and the prevention of neuronal apoptosis, stimulation of vascular regeneration, facilitation of axonal regeneration, modulation of the inflammatory response within the microenvironment, mitigation of nerve-related muscle atrophy, and enhancement of parameters associated with the human nervous system [6].

Similar finding were published by Benny et al. [19], where they compared perineural PRP injection compared to the steroids, they confirmed the short term improvement up to 12 weeks in all clinical outcomes including functional status (BCTQ FSS), symptom severity (BCTQ SSS) and CSA of the median nerve at carpal tunnel inlet.

Perineural injection of PRP has been extensively documented as a viable treatment option for CTS, demonstrating that PRP more effective than other conservative methods including steroid injection, night splinting and saline injection [20] in terms of pain reduction, improved function, and distal sensory latency improvement.Another meta-analysis demonstrated that platelet-rich plasma (PRP) injection surpasses other conservative management strategies, including wrist splinting and local injections with steroids, hyaluronidase, dextrose, and normal saline, in alleviating pain, enhancing wrist functionality and symptoms, diminishing median nerve swelling, and partially ameliorating electrophysiological parameters [21]. Others demonstrated PRP as promising therapy in short and midterm outcomes [22, 23].However, the long-term adverse side and consensus on standardization of PRP in CTS patients still need further large-scale trials.

The disparity between the results of the PRP is attributed to variations in PRP preparation techniques, which currently lack uniformity, including the volume of blood withdrawn, centrifugation duration and speed, and preactivation of platelets prior to injection [24], and the volume of PRP used, as the reported volume in different studies varied between 1 and 3 ml. It was reported that the concentration of formed elements, growth factors and cytokines in samples of PRP varied according to the centrifugation method utilized [25].

The observed improvement in the PRP group in the current study may be attributed to the swift relief of compression resulting from hydrodissection of the median nerve. However, the decreased efficacy could be explained by the the expected delayed effect of the PRP or one of the predictor factors as proposed by Shen et al., who elucidated the predictive factors contributing to the efficacy of a single PRP injection in patients with moderate carpal tunnel syndrome (CTS), specifically in terms of achieving a 50% reduction in the (VAS). Notably, lower body weight (BW), superior (SNCV), and diminished (CSA) of the median nerve (MN) were correlated with favorable outcomes during the third and sixth month follow-up visits post-PRP injection. Among these variables, BW, distal motor latency (DML), and CSA emerged as significant predictive factors of the treatment outcome.DML was identified as the most robust predictor of potential outcomes, exhibiting the strongest association (odds ratio: 0.383 at three months and 0.530 at six months). They also noted that the number of good outcomers increase from 28 patients at 3 months to 43 at six months consistent with the prolonged effect of neural regeneration [26].

Enhancements in NCV parameters were observed across all three groups throughout the four-month evaluation period. However, the PRP group demonstrated a relatively diminished degree of advancement. This observation prompted a revisit to Hakan Uzun’s study conclusion, which posits that PRP provides only transitory symptomatic relief, lacking any significant differentiations in NCV studies conducted on mild cases, thereby corroborating prior findings [27]. Indeed, several researchers have reported discrepancies between clinical symptoms and electrophysiological outcomes, as the large myelinated fibers assessed during electrophysiological examinations do not adequately reflect the small sensory fibers associated with the symptoms of CTS. Furthermore, previous investigations have indicated that electrodiagnostic measurements possess inherent limitations in forecasting the therapeutic efficacy of conservative treatments, as nerve recovery is typically a protracted process that may extend up to 18 months [20].

In the present study, notable progressive enhancements in all clinical parameters were documented at various follow-up intervals in the PRF group.The mechanism of therapeutic effect of PRF in CTS is unclear.PRF exerts its therapeutic influence through potentially multifactorial, mechanisms, such as the modulation of nociceptive Aδ and C fibers, alongside alterations in the expression of mediators implicated in neuropathic pain. Further investigation is imperative to elucidate the mechanisms by which PRF exerts its effects in CTS [8, 28].

The body of literature remains limited regarding the comparative analysis of PRF in the context of CTS. To date, this represents the inaugural research to compare (PRP) with (PRF).However, a limited number of studies have been published that compare these treatments with other conservative measures and corticosteroid injections. Gupta et al. elucidate findings from investigations on (PRF) for CTS, demonstrating notable analgesic and functional advantages, evidenced by short-term pain alleviation and enhanced functionality. However, uncertainty persists regarding long-term benefits. PRF may represent a viable alternative, particularly considering the inherent side effects associated with local steroid injections, which include adverse local reactions such as atrophy and hypopigmentation, alongside a plethora of potential systemic complications. Their review was constrained by the variability in technical parameters, encompassing probe length, active tip, needle gauge, and treatment duration. Although the output voltage has infrequently been documented in the studies reviewed, it consistently adheres to a standard output of 45 V. Ultimately, the absence of standardization in PRF parameters complicates the comparison of outcomes across diverse studies [7].

Eren Celenlioglu and colleagues [28] reported comparable enhancements in NRS and BCTQ scores with both PRF and steroid injection for a period of up to three months. Individuals who underwent PRF treatment reported significantly quicker onset of pain relief than those who received steroid injections exclusively probably due late onset of the anti-inflammatory effects of the steroid injection. The results of their study is congruent to the current study despite using mixture containing dexamethasone 8 mg and 0.5 cc of bupivacaine 0.5% targeting the hydrodissection only under the median nerve.

Similarly, Chen et al. [5]conducted a prospective, randomized, controlled, single-blinded study comparing pulsed radiofrequency (PRF) treatment of the median nerve to nocturnal splinting. The ultrasound-guided PRF cohort exhibited a more rapid onset of therapeutic effects, with a median onset time of 2 days compared to 14 days, and demonstrated a more pronounced reduction in pain at each follow-up assessment up to the 12-week. Furthermore, there was a significant enhancement in scores on the BCTQ, a reduction in the CSA of the median nerve, and improved finger pinch strength relative to the control group. The side effects associated with PRF were minimal, primarily manifesting as transient tingling at the puncture site. However, the absence of significant differences in sensory nerve conduction velocity (SNCV) engenders ongoing debate regarding the correlation between electrophysiological evaluations and the severity of symptoms or functional status scores. Despite the similarities in results, notable distinctions exist between the current study, which employed the injection of local anesthetic following PRF to standardize the integral effect of volume injection hydrodissection across all groups,. Additionally, their study did not delineate the severity of CTS in the enrolled patients. The inclusion and exclusion criteria suggest that the study likely encompassed a spectrum of CTS severity, provided that the diagnosis was substantiated by clinical symptoms and electrophysiological assessments.

In the current study hydrodissection was done in all the groups below and under the nerve to separate the nerve from the underlying tendons and overlying retinaculum. This adjustment was implemented to mitigate the hydrodissection effects across all groups uniformly.However, existing literature indicates that the inclusion of bupivacaine in conjunction with PRP may diminish the therapeutic efficacy of PRP; thus, it was excluded from the formulation and relayed on the liquid nature of PRP for hydrodissection.Indeed lack of bupivacaine might have skewed the results of PRP especially in the complication of pain during injection and was added to the limitations [29].

In the present study, only minor complications were documented across all groups, underscoring the safety of all procedures conducted over a short-term duration of up to four months but still concerns about potential side effects of steroids like skin pigmentation, tendon atrophy, hyperglycaemia and others will always be compelling to pursue other therapeutic modalities with similar efficacy [30].

### Limitations

Several limitations are evident within the study, including a brief follow-up duration; an extended follow-up period of six months is essential to thoroughly assess the long-term outcomes and complications associated with the study. Additionally, as a single-center investigation, the findings underscore the necessity for broader multicenter studies. The absence of a universally standardized protocol for the preparation of (PRP), along with the lack of platelet count measurement for each PRP sample. Furthermore, bupivacaine was excluded from the PRP in accordance with reports suggesting its potential to diminish therapeutic efficacy.

## Conclusion

The present investigation proposes that utilizing ultrasound to guide hydrodissection of the median nerve, in conjunction with local anesthetics/steroids or PRP and PRF, can be deemed efficacious modalities for addressing mild to moderate cases of carpal tunnel syndrome. Notably, short-term functional outcomes upto 4 month duration of the study were improved through the use of PRF and steroid injections; these treatments also proved superior in relieving pain compared with PRP injections.

## Supplementary Information

Supplementary Material 1.

Supplementary Material 2.

## Data Availability

The data used to support the findings of this study are available from the corresponding author upon request.

## Abbreviations

CTS: Carpal tunnel syndrome
VAS: Visual analog scale
BCTQ: Boston carpal tunnel questionnaire
NCV: Nerve conduction velocity
DML: Distal motor latency
CSA: Cross-sectional area
PRP: Platelet-rich plasma
PRF: Pulsed radiofrequency
MN: Median nerve
BW: Body weight
NRS: Numerical rating score

## References

[1] Lauder A, Mithani S, Leversedge FJ. Management of recalcitrant carpal tunnel syndrome. J Am Acad Orthop Surg. 2019;27(15):551–62. 30973521 10.5435/JAAOS-D-18-00004

[2] Padua L, Coraci D, Erra C, et al. Carpal tunnel syndrome: clinical features, diagnosis, and management. Lancet Neurol. 2016;15(12):1273–84. 27751557 10.1016/S1474-4422(16)30231-9

[3] Wang J, Hsu P, Wang KA, Chang K. Ultrasound-guided triamcinolone acetonide hydrodissection for carpal tunnel syndrome: a randomized controlled trial. Front Med. 2021;8: 742724. 10.3389/fmed.2021.742724 PMC8475784 34589506

[4] Cho H, Jang S, Lee S, et al. Effect of neural-induced mesenchymal stem cells and platelet‐rich plasma on facial nerve regeneration in an acute nerve injury model. Lancet Neurol. 2010;120(5):907–13. 10.1002/lary.20860 20422684

[5] Chen LC, Ho CW, Sun CH, Lee JT, Li TY, Shih FM, Wu YT. Ultrasound-guided pulsed radiofrequency for carpal tunnel syndrome: a single-blinded randomized controlled study. PLoS One. 2015;10(6):e0129918. 26067628 10.1371/journal.pone.0129918 PMC4466776

[6] - Sánchez M, Garate A, Delgado D, Padilla S. Platelet-rich plasma, an adjuvant biological therapy to assist peripheral nerve repair. Neural Regeneration Res. 2017;12(1):47–52. 10.4103/1673-5374.198973 PMC5319232 28250739

[7] Gupta H, Vance C, Bansal V, Siva A. A narrative review of pulsed radiofrequency for the treatment of carpal tunnel syndrome. Pain Pract. 2024;24(2):374–82. 37784211 10.1111/papr.13299

[8] Cohen BR, Soriano ET. Pulsed radiofrequency neuromodulation in interventional pain management—a growing technology. J Radiol Nurs. 2018;37(3):181–7.

[9] Schulz KF, Altman DG, Moher D. CONSORT 2010 statement: updated guidelines for reporting parallel group randomized trials. J Pharmacol Pharmacother. 2010;1(2):100–7. 21350618 10.4103/0976-500X.72352 PMC3043330

[10] Padua L, Monaco ML, Padua R, Gregori B, Tonali P. Neurophysiological classification of carpal tunnel syndrome: assessment of 600 symptomatic hands. Ital J Neurol Sci. 1997;18:145–50. 9241561 10.1007/BF02048482

[11] -Nusrat A. Diagnosis of carpal tunnel syndrome in perspective of clinical features, neurophysiological studies and high resolution ultrasound. World J Adv Res Reviews. 2020;6(3):086–96.

[12] Bland JD. A neurophysiological grading scale for carpal tunnel syndrome. Muscle Nerve. 2000;23(8):1280–3. 10918269 10.1002/1097-4598(200008)23:8<1280::aid-mus20>3.0.co;2-y

[13] Padua L, Padua R, Aprile I, Caliandro P, Tonali P. Boston carpal tunnel questionnaire: the influence of diagnosis on patient-oriented results. Neurol Res. 2005;27(5):522–4. 15978179 10.1179/016164105X17260

[14] Stark H, Amirfeyz R. Cochrane corner: local corticosteroid injection for carpal tunnel syndrome. Journal of Hand Surgery (European Volume). 2013;38(8):911–4. 24065747 10.1177/1753193413490848

[15] Moon H, Lee BJ, Park D. Change to movement and morphology of the median nerve resulting from steroid injection in patients with mild carpal tunnel syndrome. Sci Rep. 2020;10(1): 15607. 32973181 10.1038/s41598-020-72757-2 PMC7515891

[16] -Yamanaka Y, Tajima T, Tsujimura Y, Kosugi K, Mano Y, Zenke Y, et al. Molecular and clinical Elucidation of the mechanism of action of steroids in idiopathic carpal tunnel syndrome. J Bone Jt Surg. 2021;103(19):1777–87. 10.2106/JBJS.20.02096 34398862

[17] -Cage ES, Beyer JJ, Ebraheim NA. Injections for treatment of carpal tunnel syndrome: A narrative review of the literature. J Orthop. 2023;37:81–5. 36974095 10.1016/j.jor.2023.02.011 PMC10039115

[18] Roh YH, Hwangbo K, Gong HS, Baek GH. Comparison of ultrasound-guided versus landmark-based corticosteroid injection for carpal tunnel syndrome: a prospective randomized trial. J Hand Surg Am. 2019;44(4):304–10. 30947825 10.1016/j.jhsa.2019.02.007

[19] Benny R, Venkataraman S, Chanu AR, Singh U, Kandasamy D, Lingaiah R. A randomized controlled trial to compare the effect of Ultrasound–Guided, Single–Dose Platelet–Rich plasma and corticosteroid injection in patients with carpal tunnel syndrome. J Int Soc Phys Rehabilitation Med. 2022;5(3):90.

[20] Dong C, Sun Y, Qi Y, et al. Effect of platelet-rich plasma injection on mild or moderate carpal tunnel syndrome: an updated systematic review and meta-analysis of randomized controlled trials. Biomed Res Int. 2020;2020:5089378. 33274213 10.1155/2020/5089378 PMC7683131

[21] Jiang J, Xing F, Luo R, Liu M. Effectiveness of platelet-rich plasma for patients with carpal tunnel syndrome: a systematic review and meta-analysis of current evidence in randomized controlled trials. Front Pharmacol. 2022;13:834213. 35571114 10.3389/fphar.2022.834213 PMC9092282

[22] Malahias MA, Chytas D, Mavrogenis AF, Nikolaou VS, Johnson EO, Babis GC. Platelet-rich plasma injections for carpal tunnel syndrome: a systematic and comprehensive review. Eur J Orthop Surg Traumatol. 2019;29:1–8. 30022241 10.1007/s00590-018-2278-8

[23] Catapano M, Catapano J, Borschel G, Alavinia SM, Robinson LR, Mittal N. Effectiveness of platelet-rich plasma injections for nonsurgical management of carpal tunnel syndrome: a systematic review and meta-analysis of randomized controlled trials. Arch Phys Med Rehabil. 2020;101(5):897–906. 31821797 10.1016/j.apmr.2019.10.193

[24] Lai C, Li T, Lam KHS, et al. The long-term analgesic effectiveness of platelet-rich plasma injection for carpal tunnel syndrome: a cross-sectional cohort study. Pain Med. 2022;23(7):1249–58. 35043941 10.1093/pm/pnac011

[25] -Pochini AC, Antonioli E, Bucci DZ, Sardinha LR, Andreoli CV, Ferretti M, Ejnisman B, Goldberg AC, Cohen M. Analysis of cytokine profile and growth factors in platelet-rich plasma obtained by open systems and commercial columns. Einstein (Sao Paulo). Einstein (Sao Paulo). 2016;14(3):391–7. 27759829 10.1590/S1679-45082016AO3548 PMC5234752

[26] Shen YP, Li TY, Chou YC, Chen LC, Wu YT. Outcome predictors of platelet-rich plasma injection for moderate carpal tunnel syndrome. Int J Clin Pract. 2021;75(10): e14482. 34107143 10.1111/ijcp.14482

[27] Uzun H, Bitik O, Uzun Ö, Ersoy US, Aktaş E. Platelet-rich plasma versus corticosteroid injections for carpal tunnel syndrome. J Plast Surg Hand Surg. 2017;51(5):301–5. 27921443 10.1080/2000656X.2016.1260025

[28] Celenlioglu AE, Unal-Artık HA, Guler G. Comparison of ultrasound-guided pulsed radiofrequency versus steroid injection in the treatment of carpal tunnel syndrome. Ir J Med Sci. 2022;191(6):2751–7. 35129753 10.1007/s11845-022-02923-0

[29] Bausset O, Magalon J, Giraudo L, Louis ML, Serratrice N, Frere C, Magalon G, Dignat-George F, Sabatier F. Impact of local anaesthetics and needle calibres used for painless PRP injections on platelet functionality. Muscles Ligaments Tendons J. 2014;4(1):18–23. 24932442 PMC4049644

[30] Hsu PC, Liao KK, Lin KP, et al. Comparison of corticosteroid injection dosages in mild to moderate idiopathic carpal tunnel syndrome: a randomized controlled trial. Arch Phys Med Rehabil. 2020;101(11):1857–64. 32682938 10.1016/j.apmr.2020.06.018

